# Reverse Versus Modified Judet Approach for Extra‐Articular Glenoid Neck and Scapular Body Fractures: A Retrospective Study

**DOI:** 10.1111/os.70357

**Published:** 2026-06-03

**Authors:** Qianxi Wang, Zehao Guo, Jingtian Shi, Mingfan Wu, Huai Jiang, Cong Sui, Yukang Que, Peng He, Yong Hu, Shenglin Xu

**Affiliations:** ^1^ Department of Orthopedics The First Affiliated Hospital of Anhui Medical University Hefei Anhui China; ^2^ Department of Musculoskeletal Oncology The First Affiliated Hospital of Sun Yat‐Sen University Guangzhou Guangdong China; ^3^ Department of Orthopedics Lu'an Hospital of Anhui Medical University Lu'an Anhui China

**Keywords:** clinical outcomes, modified Judet, reverse Judet, scapular fracture

## Abstract

**Objective:**

The reverse Judet approach is a modified technique for scapular fractures, designed to reduce tissue damage and minimize surgical morbidity. However, its comparative efficacy and safety relative to the modified Judet approach have not been well established. Therefore, this study aimed to (1) evaluation of the safety and feasibility of reverse Judet approach for scapular fractures and (2) comparing the advantages and disadvantages of reverse Judet and modified Judet approaches in perioperative indicators and postoperative functional recovery.

**Methods:**

A retrospective analysis was conducted on 40 patients with extra‐articular glenoid neck or scapular body fractures admitted between July 2021 and September 2023. The cohort comprised 18 cases in the reverse Judet group and 22 cases in the modified Judet group. Baseline data, surgical parameters (incision length, flap area, operative time, intraoperative blood loss), functional outcomes (DASH score, Constant–Murley score), and complications were recorded.

**Results:**

There were no significant differences in baseline characteristics between the two groups. The reverse Judet group showed significantly better outcomes compared to the modified Judet group in terms of incision length (15.2 ± 1.3 vs. 20.1 ± 1.0 cm, *p* < 0.001) and flap area (49.5 ± 6.2 cm^2^ vs. 67.4 ± 4.7 cm^2^, *p* < 0.001). Although not statistically significant, the reverse Judet group tended to have shorter operative times (100.2 ± 20.1 113.4 ± 21.2 min, *p* = 0.053) and less intraoperative blood loss (136.7 ± 36.0 vs. 155.2 ± 26.9 mL, *p* = 0.051). Functional assessments showed no significant differences in DASH scores (7.8 ± 2.7 vs. 9.5 ± 4.1, *p* = 0.156) or Constant–Murley scores (84.3 ± 5.4 vs. 84.1 ± 4.6, *p* = 0.929) between groups. Neither group experienced nonunion, implant‐related complications, or neurovascular injury.

**Conclusion:**

The reverse Judet approach offers benefits such as smaller incisions and less tissue damage while providing adequate exposure for fracture reduction and fixation. Functional recovery is similar to that of the modified Judet approach. Its minimally invasive nature and better esthetic results make it a more comprehensive surgical option for posterior shoulder issues. Although this study did not find an increased risk of flap complications, larger studies are necessary to further improve flap design.

## Graphical abstract

This study shows that the reverse Judet approach provides functional results similar to the modified Judet approach in treating scapular fractures. Importantly, it greatly reduces the size of the incision and limits soft tissue dissection, which decreases surgical trauma. Therefore, the reverse Judet approach is a safe and effective option for treating scapular fractures.

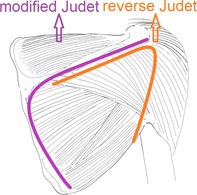

## 1 Introduction

Scapula fractures account for 1% of all fractures and 5% of all shoulder girdle fractures [1]. Due to its deep location and enveloped by muscles, polytrauma, including concomitant cranial and thoracic injuries caused by high energy, is common. The necessity to deal with the prior injuries left the fracture treated with benign neglect. However, scapular fractures resulted in a defunct dynamic stabilizer of the humerus and shoulder complex [2].

Surgical treatment later turned out to be a better option and an undercurrent of open reduction and internal fixation (ORIF) in selected patients has emerged [3]. The original approach required a boomerang‐shaped incision along the spine and the medial scapula, followed by the extensive peeling of infraspinatus to expose the fossa and glenoid for reduction and fixation [4]. The reconstruction of the rotator cuff and the risk of iatrogenic nerve injuries contributed to the compromised functional recovery [5]. Modified Judet approach, which was developed to arrive at the bony landmarks via the intermuscular space, was proven to attain superior functional outcomes [6, 7]. According to the three‐dimensional computerized mapping technique, the inferior glenoid is the most frequently involved site of the fractures. And the axillary border is prone to severe displacement inflicted by deforming forces [8]. Thus, a safe and direct approach to the lateral border is of paramount importance for manipulation. Typically, a vertical lateral incision allows successful placement of a plate with limited exposure for fractures of a simple pattern; however, when the fracture line is located in the scapular neck or accumulates in the scapular body, which is depicted as comminuted and requires additional reduction, the exposure is insufficient [9]. The Reverse Judet approach, which forms a full‐thickness fasciocutaneous flap with a base located medially, would theoretically have a better visual field of lateral structures and less trauma [10, 11, 12].

We performed the present study to (1) evaluate the feasibility and safety of the reverse Judet approach for extra‐articular glenoid neck and scapular body fractures and (2) compare the clinical outcomes between patients treated by the reverse Judet approach and the modified Judet approach, with a minimum follow‐up of 12 months.

## 2 Materials and Methods

### 2.1 Patient

The inclusion criteria were (i) age between 18 and 70 years; (ii) a minimum postoperative follow‐up of 12 months; and (iii) availability of complete perioperative and follow‐up data. The exclusion criteria were (i) old fracture of the scapula; (ii) open scapular fracture; (iii) pathological scapular fracture; and (iv) concomitant severe craniocerebral or brachial plexus injury. Patients with displaced extra‐articular fractures of the glenoid neck or scapular body (AO/OTA types 14A3 and 14C1) who underwent ORIF between July 2021 and September 2023 were screened for eligibility. This retrospective cohort study was approved by the Clinical Research Ethics Committee of the First Affiliated Hospital of Anhui Medical University (Approval No. PJ2025‐10‐76). Surgical indications for ORIF included: (i) displacement greater than 20 mm; (ii) angulation greater than 40°; (iii) combination of displacement greater than 15 mm and angulation greater than 30°; (iv) glenoid polar angle less than 22°; and (v) more than two ruptures of the superior suspensory shoulder complex. From July 2021 to September 2023, 98 scapular fractures were treated. All 83 patients had scapular neck and scapular body fractures. Three experienced orthopedic surgeons initially classified all 83 patients with scapular neck and body fractures using the AO/OTA classification system [13]. Later, 59% (58 patients) were excluded due to loss to follow‐up or incomplete data before reaching the minimum follow‐up period. In the end, 41% (40 patients) with 14A3 and 14C1 fractures that met surgical indications were included in the analysis. According to the surgeon's experience, both the modified Judet technique and the reverse Judet technique had favorable therapeutic effects, and either surgical method was randomly chosen. Patients who met the inclusion criteria (*n* = 40) were invited for a secondary functional evaluation. A total of 18 patients were treated with the reverse Judet approach; 22 cases were treated with the modified Judet approach. Demographics of the patients are presented in Table 1.

**TABLE 1:** Demographics of the patients.

	Reverse Judet	Modified Judet	*p*
Age (years)	46.7 ± 10.2	52.7 ± 10.8	0.081
Gender			0.626
Male	16	19	
Female	2	3	
BMI (kg/m^2^)	24.5 ± 3.5	23.4 ± 2.0	0.216
AO/OTA classification			0.919
A3	12	14	
C1	6	8	
Concomitant injury			
Chest injury	4	5	0.937
Clavicle fracture	5	5	0.696

### 2.2 Outcome Measures

#### 2.2.1 Surgical Parameters

*Incision length*: The length of curved skin incisions was recorded and measured with sutures during surgery or postoperatively, recorded in centimeters (cm).

*Flap area*: Calculated postoperatively by importing surgical field photographs into ImageJ software (National Institutes of Health, Bethesda, MD, USA), reported in square centimeters (cm^2^).

*Operative time*: Defined as the time from skin incision to wound closure, retrieved from anesthetic records, recorded in minutes (min).

*Intraoperative blood loss*: Estimated by the anesthesiologist based on suction volume and gauze weight, retrieved from anesthetic records, recorded in milliliters (mL).

#### 2.2.2 Functional Outcomes (Assessed at the Latest Follow‐Up, Minimum 12 Months)

*Constant–Murley score* [14]: A physician‐administered score (range 0–100) evaluating pain, activities of daily living, active range of motion, and strength. Higher scores indicate better function.

*Disabilities of the arm, shoulder, and hand (DASH) score* [15]: A patient‐reported questionnaire (range 0–100) assessing upper extremity disability and symptoms. Lower scores indicate less disability.

#### 2.2.3 Complications

Recorded throughout the follow‐up period, including but not limited to surgical site infection, wound dehiscence, neurovascular injury, implant failure, nonunion, and shoulder instability.

### 2.3 Surgical Technique

We performed clavicle fracture fixation before the scapula. Each patient with an ipsilateral clavicle fracture was positioned in a floppy lateral position that permits changing to either an anterior or posterior stage when necessary.

#### 2.3.1 Group I Modified Judet Approach

The incision design and the steps detailing access to the fracture line were similar to those described in a previously published study [16]. The principle of deltoid detachment was in concord with the reverse Judet group [17].

#### 2.3.2 Group II Reverse Judet Approach (Figure 1)

**FIGURE 1:**
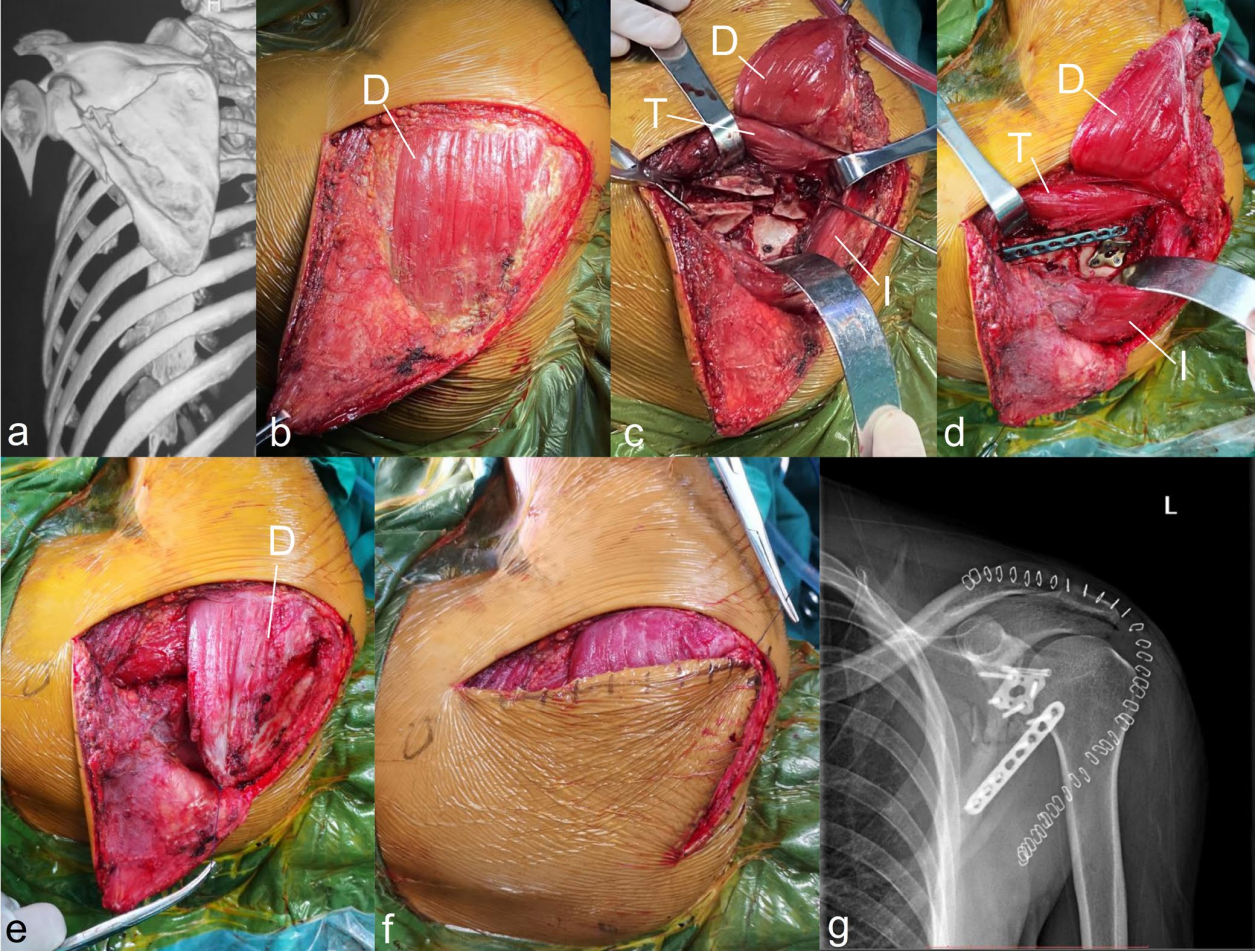
A 57‐year‐old male patient underwent treatment via the reverse Judet approach. (a) Preoperative 3D CT scan revealed a comminuted fracture of the scapular neck; (b–f) after muscle dissection, internal fixation and muscle flap suturing were performed (D: deltoid muscle, I: infraspinatus muscle, T: teres minor muscle); (g) postoperative X‐ray demonstrating restoration of normal anatomical structures.

A reverse Judet approach was conducted by creating an incision that started medially and extended along the lateral 2/3 of the scapular spine. It curved distally along the lateral border of the scapula to the posterolateral corner of the acromion [11]. The transverse incision could start less medially and span further laterally in muscular individuals, which allowed medial extension or exposure of the scapular neck if necessary.

The full‐thickness fasciocutaneous flap was created and reflected medially to expose the posterior muscles. An acute apex angle should be avoided to preserve the blood supply. The dissection was taken down to the deltoid fascia, which was divided in line with the muscular fiber at the inferior edge. The plane between the posterior deltoid and infraspinatus was developed through blunt dissection. The deltoid was then retracted cephalad with arms widely abducted, revealing the combined fascia overlying the external rotators. Alternatively, in patients with a stout figure, sharp dissection was executed 0.5 cm distal from the origin of the partial deltoid for mobilization and further reconstruction. The interval between the infraspinatus and the teres minor was identified and the muscles were retracted proximally and distally for the visualization of the lateral border and posterior glenoid. The suprascapular nerve, which coursed via the spinoglenoid notch to innervate the supraspinatus and infraspinatus, was protected from aggressive retraction. The circumflex scapular artery located at the lateral border of the scapula and approximately 5 cm below the inferior glenoid was cauterized in most cases.

The bony fragments were temporarily fixed by K‐wires. After fracture reduction, locking plates or pre‐bent reconstruction plates were used to fix the glenoid neck and lateral border. The stability of the fixation was examined by passive movement of the shoulder. After the electrocoagulation of bleeding flecks, a drainage tube was placed in the intermuscular space. The deltoid was then reattached with 2.0 non‐absorbable sutures. And the wound was closed in layers.

### 2.4 Postoperative Management

Rehabilitation was initiated immediately on the first or second postoperative day, with passive exercises, once the pain had been slightly alleviated. Under the protection of a forearm sling, small pendulum movements were performed with a gradient increase in range of motion. Active exercises were encouraged after 2 weeks and the focus was placed on regaining a full active range of motion after 1 month, with X‐ray confirmed bone healing. The postoperative follow‐up protocol involves reviewing anteroposterior and lateral x‐rays of the shoulder at 1, 3, 6, and 12 months after surgery. Follow‐up may be stopped if radiographic evidence shows fracture healing and the patient reports no restrictions in daily activities. If any abnormalities are detected during follow‐up, monitoring should continue. The functional outcomes were evaluated with the DASH questionnaire and the Constant–Murley score at the latest follow‐up.

### 2.5 Statistical Analysis

SPSS software version 25.0 (IBM, Armonk, New York) was used for all statistical analyses. Data with normal distribution was expressed as mean ± standard deviation and analyzed using a *t*‐test and the counting data was analyzed by *χ* ^2^. A value of *p* < 0.05 was considered significant.

## 3 Results

### 3.1 Comparison of Intraoperative Metrics

The reverse Judet approach demonstrated comparable intraoperative metrics to the modified Judet approach in managing extra‐articular glenoid neck and scapular body fractures. Operative duration (100.2 ± 20.1 vs. 113.4 ± 21.2 min, *p* = 0.053) and intraoperative blood loss (136.7 ± 36 vs. 155.2 ± 26.9 mL, *p* = 0.051) trended toward shorter duration and reduced blood loss in the reverse Judet group, though these differences did not reach statistical significance. Notably, the reverse Judet approach was associated with a significantly smaller flap area (49.5 ± 6.2 vs. 67.4 ± 4.7 cm^2^, *p* < 0.001) and shorter incision length (data not reported for reverse Judet vs. 20.1 ± 1.0 cm for modified Judet, *p* < 0.001), highlighting technical advantages in tissue preservation and surgical invasiveness (Table 2).

**TABLE 2:** Patient outcomes.

	Reverse Judet	Modified Judet	*p*
Operative metrics			
Operative duration (min)	100.2 ± 20.1	113.4 ± 21.2	0.053
Blood loss (mL)	136.7 ± 36	155.2 ± 26.9	0.051
Incision length (cm)	15.2 ± 1.3	20.1 ± 1.0	< 0.001
Flap area (cm^2^)	49.5 ± 6.2	67.4 ± 4.7	< 0.001
Functional score			
DASH score	7.8 ± 2.7	9.5 ± 4.1	0.156
Constant–Murley score	84.3 ± 5.4	84.1 ± 4.6	0.929

### 3.2 Comparison of Functional Recovery Outcomes

**FIGURE 2:**
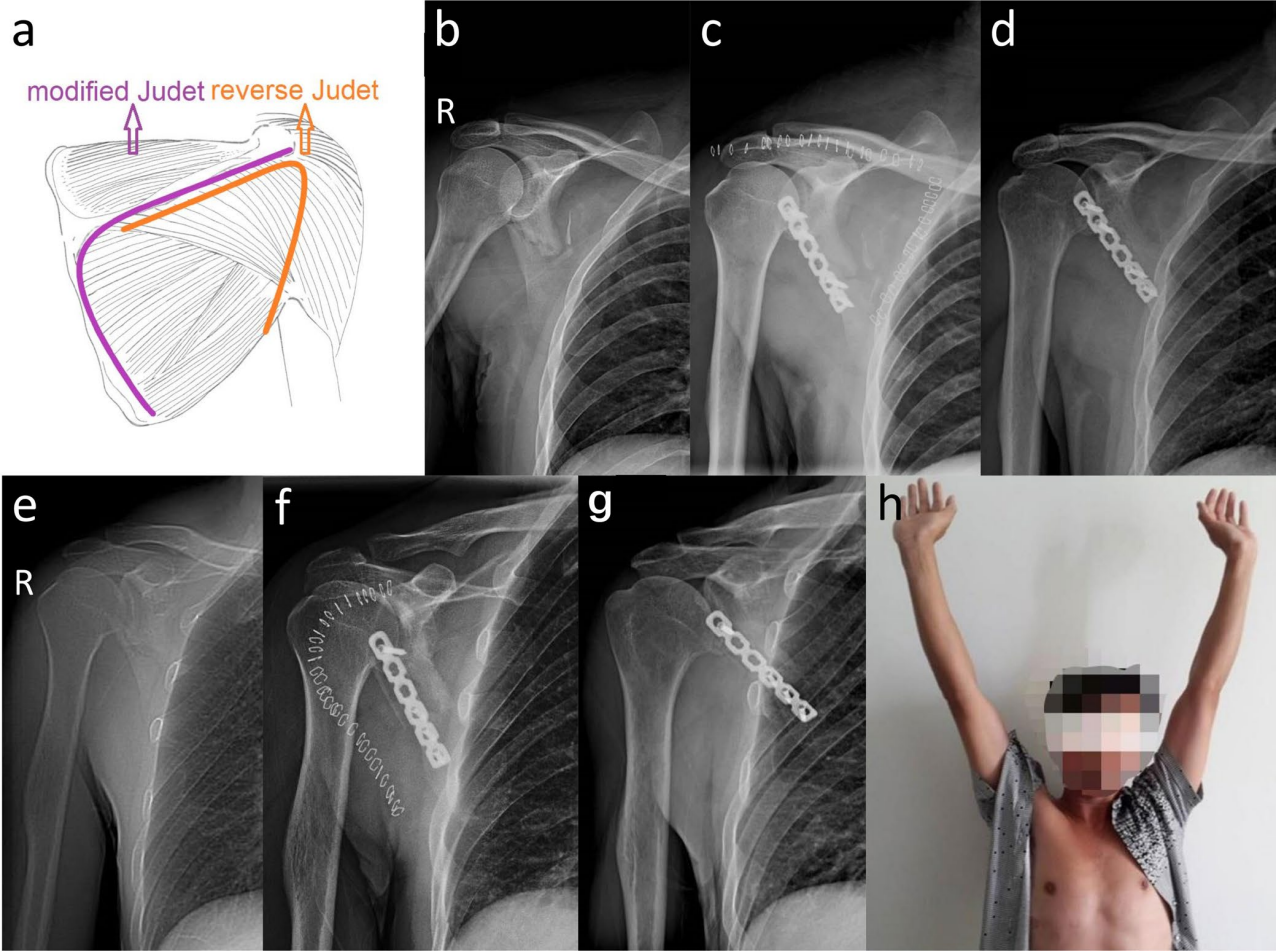
Follow‐up imaging of the modified Judet and reverse Judet approaches for scapular fractures. (a) Schematic illustration of the surgical incisions for the modified Judet and reverse Judet approaches; (b–d) a 57‐year‐old male patient was treated with the modified Judet approach. Preoperative scapular imaging (b). Immediate postoperative scapular imaging (c). Follow‐up scapular imaging at 14 months postoperatively (d); (e‐h). A 54‐year‐old male patient was treated with the reverse Judet approach. Preoperative scapular imaging (e). Immediate postoperative scapular imaging (f). Follow‐up scapular imaging at 12 months postoperatively (g). Functional outcome photograph of the patient showing shoulder function recovered at 12 months follow‐up (h).

Functional restoration, as assessed by the Constant–Murley score (84.3 ± 5.4 vs. 84.1 ± 4.6, *p* = 0.929) and DASH score (7.8 ± 2.7 vs. 9.5 ± 4.1, *p* = 0.156), showed no significant differences between the two groups. These results suggest equivalent functional recovery and pain resolution outcomes (Figure 2). However, the reverse Judet group can start rehabilitation exercises as early as 1 week after the operation, while the modified Judet group can only start rehabilitation exercises 3–4 weeks after the operation (Table 2).

### 3.3 Postoperative Complications

No intraoperative complications (e.g., neurovascular injury) or postoperative complications (including infection, delayed wound healing, or shoulder instability) were observed in either group [18, 19]. Two patients undergoing reverse Judet procedures developed tension blisters at the distal apex of the skin margin 1 day postoperatively due to improper flap incision design, where the angle between the lateral border of the scapula and the scapular spine incision was less than 45 degrees. The blisters resolved and the wounds healed by 2 weeks postoperatively, with no skin margin necrosis observed in either case. The absence of complications may be attributed to the limited sample size, which reduces the statistical power to detect rare adverse events [5]. To comprehensively evaluate the safety profiles of both techniques, future studies with larger cohorts are warranted to assess the severity and subtype‐specific incidence of complications.

## 4 Discussion

This retrospective comparative study demonstrates that the reverse Judet approach is a safe and feasible alternative to the modified Judet approach for the surgical management of extra‐articular glenoid neck and scapular body fractures. The reverse Judet approach resulted in significantly smaller surgical incisions and reduced flap areas compared to the modified Judet approach. Furthermore, it proved to be non‐inferior regarding operative duration and blood loss, while achieving comparable functional outcomes at the minimum 12‐month follow‐up. Crucially, no major neurovascular complications or implant‐related failures were observed in either treatment group.

### 4.1 Feasibility and Safety of the Reverse Judet Approach

In this study, the reverse Judet approach was performed along muscle planes (e.g., the infraspinatus‐teres minor interval), avoiding the extensive dissection or transection of rotator cuff muscles like the infraspinatus required for broad exposure in the traditional Judet approach [20]. This significantly reduced iatrogenic damage to muscle tissue during surgery. Simultaneously, this approach optimizes incision design (along the scapular spine and axillary border), maximizing surgical exposure while drastically minimizing the area of skin flap mobilization. This reduces soft tissue trauma and bleeding. This precise, limited dissection maximally preserves the neural innervation and blood supply of the rotator cuff muscles, establishing the biological foundation for their early active contraction and functional rehabilitation [21]. Furthermore, the intraoperative framework stabilization concept prioritizes restoring stability to the lateral column of the scapula and the scapular spine, providing reliable mechanical support for early shoulder joint mobilization. The clinical benefits of early rehabilitation are multifaceted [22]. Functionally, early passive and active movements under protection effectively prevent soft tissue adhesions and capsular contracture around the shoulder joint, which is crucial for maintaining and restoring shoulder range of motion—particularly, external rotation and elevation [23]. In terms of recovery progression, earlier functional exercises promote local blood circulation, accelerate swelling resolution and tissue healing, and reduce the risk of complications.

### 4.2 Efficacy and Potential Indications of the Reverse Judet Approach

**FIGURE 3:**
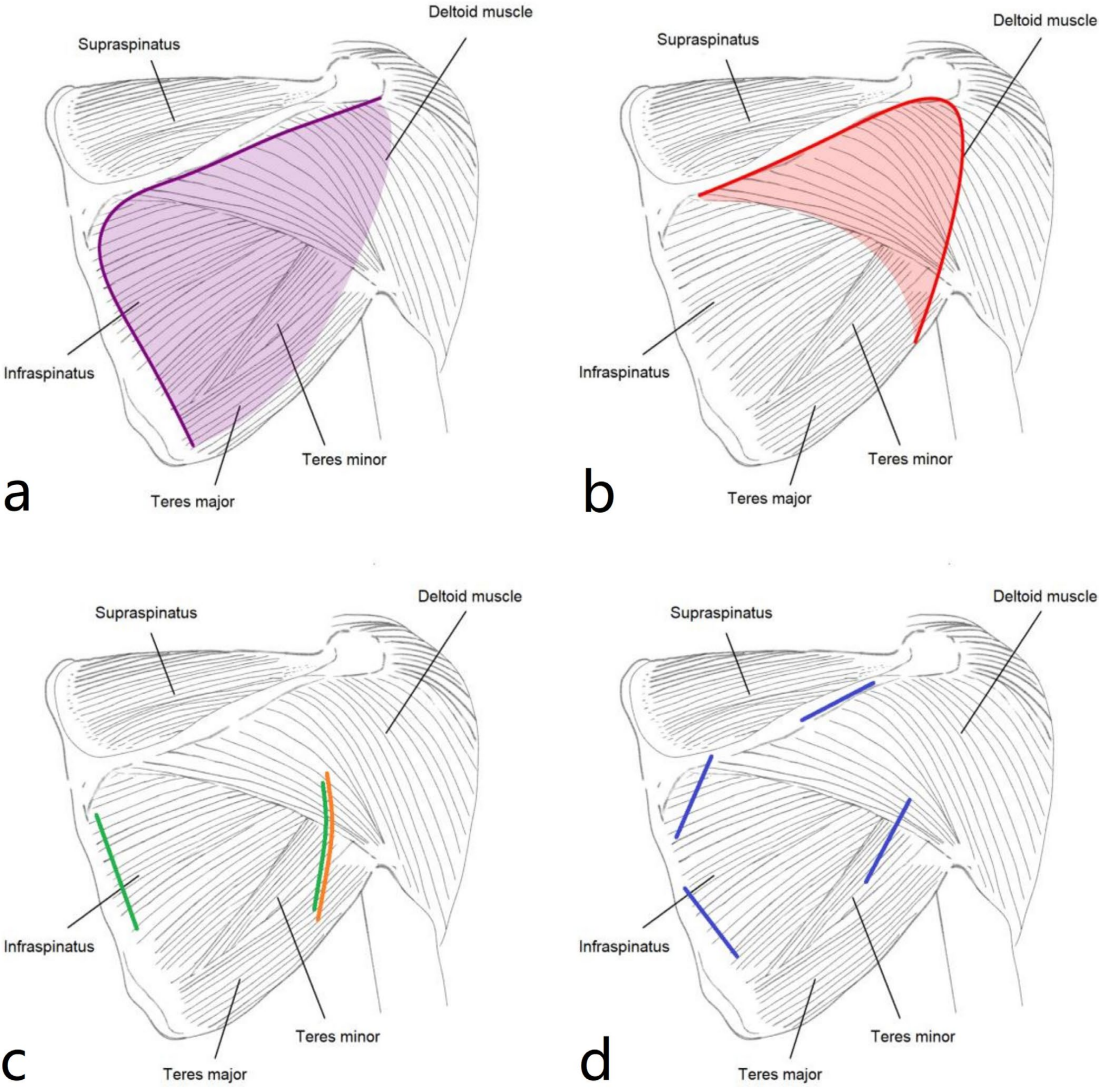
Illustration comparing surgical incision approaches for the posterior scapular approach. (a) The reverse Judet incision formed a triangular flap with a concave bottom line; (b) the modified Judet incision formed a triangular flap with a convex base; (c) the single straight incision is primarily made along the lateral border of the scapula (orange marker). If the fracture is complex, an additional incision may be made along the medial border of the scapula (green marker); (d) minimally invasive multiple incision techniques for the scapula typically involve two to three short incisions.

The classic Judet approach employs a large inverted “L” incision along the scapular spine and medial border, necessitating extensive retraction of the subscapularis muscle for exposure [24]. While providing ample visualization, it causes significant damage to the soft tissue. The modified Judet approach features a similar incision but enters through the subscapularis‐teres minor muscle gap, minimizing muscle retraction and balancing injury control with exposure (Figure 3a) [17]. The single straight incision causes less trauma than the reverse Judet approach and allows direct management of lateral margin fractures, but it offers limited exposure and presents challenges with complex fractures (Figure 3c) [25]. Minimally invasive multi‐incision techniques cause minimal trauma and scarring, but this approach provides limited surgical visualization and is technically demanding (Figure 3d) [26]. The reverse Judet approach offers three key advantages: First, optimized incision and exposure. Its curved incision closely follows the lateral column of the scapula—a critical area for fracture reduction—enabling thorough exposure with a shorter incision length and smaller flap area (significantly superior to the modified Judet group in this study), particularly suitable for complex fractures extending into the body [27, 28, 29]. Second, enhanced precision in muscle space manipulation. While also accessing the subscapularis‐infraspinatus and infraspinatus‐teres minor muscle spaces, this approach allows more physiologically aligned retraction of the infraspinatus muscle after medial flap elevation, potentially reducing the risk of neuromuscular injury from excessive traction (Figure 3b). Third, the potential for early functional recovery. Due to more limited soft tissue dissection and reduced vascular disruption, patients experience less postoperative pain, creating favorable conditions for initiating rehabilitation exercises early. However, this approach offers limited exposure of the scapular spine margin and is not suitable for fracture types requiring direct reduction of the medial column.

### 4.3 Limitations and Strengths

However, this study is limited by its small sample size (reverse Judet group *n* = 18, modified Judet group *n* = 22). This limitation directly results in insufficient statistical power, potentially obscuring true differences between groups in certain clinically relevant indicators (e.g., subtle variations in specific complications) while also reducing the ability to detect rare adverse events (e.g., specific nerve injuries or wound healing issues). The strengths of this study include the use of objective and quantifiable surgical metrics, such as incision length and flap area, combined with validated functional outcome measures to compare the efficacy of the two surgical approaches. Furthermore, all procedures were performed by experienced orthopedic surgeons, and a standardized postoperative rehabilitation protocol was followed, which enhanced the internal validity and clinical relevance of the findings. Given these considerations, future research should focus on conducting large‐scale, multicenter prospective cohort studies or randomized controlled trials. Expanding sample sizes will provide stronger validation of the reverse Judet approach's efficacy and safety, enabling precise evaluation of its potential advantages in promoting early rehabilitation and long‐term functional preservation. This will generate higher‐level evidence supporting the standardized clinical adoption of this minimally invasive technique.

## 5 Conclusion

The reverse Judet approach demonstrates non‐inferior clinical efficacy compared to the modified Judet technique for extra‐articular glenoid neck and scapular body fractures. It offers advantages in reduced incision length and flap area, with comparable functional outcomes. While both approaches showed satisfactory safety profiles, larger prospective studies are required to validate these findings and further elucidate the long‐term benefits.

## Author Contributions

**Qianxi Wang:** writing – original draft, investigation, formal analysis, visualization. **Yukang Que:** validation, data curation. **Yong Hu:** funding acquisition, supervision. **Jingtian Shi:** data curation, resources. **Zehao Guo:** conceptualization, methodology, writing – review and editing. **Shenglin Xu:** funding acquisition, supervision. **Peng He:** funding acquisition, supervision. **Cong Sui:** project administration, validation. **Mingfan Wu:** software, validation. **Huai Jiang:** project administration, investigation.

## Funding

This work was supported by the Major Scientific Research Projects of the Health Commission of Anhui Province (Grant AHWJ2023A10008); the Research Fund of Anhui Institute of Translational Medicine (Grant 2022zhyx‐C34); the Program for Upgrading Basic and Clinical Collaborative Research of Anhui Medical University (Grant 2023xkjT031); the Major Project of Scientific Research of the Anhui Provincial Department of Education (Grant 2024AH040116); and the Research Fund Project of Anhui Institute of Translational Medicine (Grant 2025zh‐07).

## Disclosure

All authors listed meet the authorship criteria according to the latest guidelines of the International Committee of Medical Journal Editors. All authors are in agreement with the manuscript.

## Ethics Statement

This clinical research protocol strictly adheres to ethical standards. Its scientific validity and protection of subject rights have been reviewed and approved by the Clinical Research Ethics Committee of the First Affiliated Hospital of Anhui Medical University (Approval No. PJ2025‐10‐76). The entire implementation process of this study will strictly comply with the ethical principles established by the World Medical Association's Declaration of Helsinki, particularly the version revised at the 64th World Medical Association General Assembly in Fortaleza, Brazil, in 2013.

## Conflicts of Interest

The authors declare no conflicts of interest.

## Data Availability

The data that support the findings of this study are available on request from the corresponding author. The data are not publicly available due to privacy or ethical restrictions.
